# Pre-procedural predictors of left atrial low-voltage zones in patients undergoing catheter ablation of atrial fibrillation

**DOI:** 10.1371/journal.pone.0266939

**Published:** 2022-04-12

**Authors:** Takenori Ikoma, Yoshihisa Naruse, Yutaro Kaneko, Tomoaki Sakakibara, Taro Narumi, Makoto Sano, Satoshi Mogi, Kenichiro Suwa, Hayato Ohtani, Masao Saotome, Tsuyoshi Urushida, Yuichiro Maekawa

**Affiliations:** Division of Cardiology, Internal Medicine III, Hamamatsu University School of Medicine, Hamamatsu, Japan; Ohio State University, UNITED STATES

## Abstract

Pulmonary vein isolation has become a cornerstone treatment for catheter ablation of atrial fibrillation (AF). Recent reports show that additional ablation targeting low-voltage zones reduces AF recurrence. However, the pre-procedural predictors of low-voltage zones remain elusive. We retrospectively enrolled 359 patients (mean age 63.7 ± 10.8 years; 73 females; and 149 had persistent atrial fibrillation) who underwent catheter ablation for AF and left atrial (LA) voltage mapping during sinus rhythm or atrial pacing. Low-voltage zones were defined as area of > 5 cm^2^ with a bipolar electrogram amplitude of < 0.50 mV. Overall, 51 (14.2%) patients had low-voltage zones. Patients with low-voltage zones were older (67.9 ± 9.9 vs. 63.0 ± 10.8 years; *P* = 0.003), predominantly female (33.3% vs. 18.2%; *P* = 0.013), had higher prevalence of dilated cardiomyopathy (DCM) (11.8% vs. 1.6%; *P* = 0.002) and hypertrophic cardiomyopathy (HCM) (9.8% vs. 2.6%; *P* = 0.025), and had larger LA volumes (153.6 ± 46.4 vs. 117.7 ± 67.8 mL; *P* < 0.001) than those without low-voltage zones. Multivariate logistic regression analysis revealed that age (OR 1.060; 95% CI 1.022–1.101, *P* = 0.002), female sex (OR 2.978; 95% CI 1.340–6.615, *P* = 0.007), DCM (OR 8.341; 95% CI 1.381–50.372, *P* = 0.021), HCM (OR 5.044; 95% CI 1.314–19.363, *P* = 0.018), persistent AF (OR 4.188; 95% CI 1.928–9.100, *P* < 0.001), and larger LA volume (OR 3.215; 95% CI 1.378–7.502, *P* = 0.007) were independently associated with the presence of low-voltage zones. Patient age, female sex, DCM, HCM, persistent AF and larger LA volume may predict the presence of low-voltage zones and could be useful in selecting the appropriate ablation strategy for AF.

## Introduction

Atrial fibrillation (AF) is the most common form of arrhythmia in clinical practice [1] and its presence increases the risk of cerebrovascular accident, heart failure, and all-cause death [2]. Furthermore, the prevalence of AF has increased in recent years [3]. Catheter ablation is an effective mainstream treatment for rhythm control in patients with AF [4]. Currently, pulmonary vein isolation (PVI) has become the cornerstone of catheter ablation treatments. Although PVI is effective, AF recurrence after ablation and additional required procedures remain unresolved, especially in patients with persistent AF [5–8]. However, several procedural approaches beyond PVI during catheter ablation have also been advocated to solve these problems.

Atrial remodeling, such as atrial fibrosis and scarring, plays an important role in the pathogenesis of AF [9–11]. Low-voltage zones (LVZs) are known to be involved in the remodeling process; these can be found in patients with paroxysmal and persistent AF (10% and 35%, respectively) [12, 13]. Recently, additional ablation of LVZs was shown to reduce AF recurrence after catheter ablation [13, 14]. Although balloon-based ablations with the Cryoballoon (Arctic Front Advance, Medtronic, Minneapolis, MN), hot balloon (Satake Hot balloon, Toray, Tokyo, Japan), or laser balloon (HeartLight, CardioFocus, Marlborough, MA) are generally only available for PVI, radiofrequency open-irrigated catheters can perform substrate modification of LVZ beyond PVI. Therefore, the presence of LVZ determines the selection of ablation devices before ablation. However, pre-procedural predictors of LVZ remain elusive.

In this study, we investigated the pre-procedural predictors of LVZ in patients with AF.

## Methods

### Study subjects

We retrospectively enrolled 359 consecutive patients who underwent catheter ablation for AF and left atrial (LA) voltage mapping during sinus rhythm or atrial pacing using 20-pole circular mapping catheter at the Hamamatsu University Hospital between March 2017 and March 2020, using a prospectively collected ablation database (Hamamatsu EPS registry). Patients were eligible if they were aged 18 years or older. Patients who could only obtain left atrial potentials during AF were excluded. The primary endpoint was the presence of LVZ detected using electro-anatomical voltage mapping during the ablation procedure. Patients were divided into two groups according to the presence or absence of LVZ, and pre-procedural parameters were compared between the two groups. Data on age, sex, body mass index, comorbid diseases, and medications were collected. Paroxysmal AF (terminated within 7 days) and persistent AF (lasting over 7 days) were defined according to the current guidelines [1]. We considered the patients as cardiomyopathy if the patients had following conditions: 1) left ventricular wall thickness of > 15 mm, 2) right ventricular dilatation, and 3) low LVEF (< 50%). In patients with suspected cardiomyopathy, cardiac computed tomography (CT) was performed to distinguish ischemic cardiomyopathy from non-ischemic cardiomyopathy (NICM) before the catheter ablation. NICM was classified into dilated cardiomyopathy (DCM), hypertrophic cardiomyopathy (HCM), cardiac amyloidosis, cardiac sarcoidosis, myocarditis, valvular heart disease, drug-induced cardiomyopathy, arrhythmogenic right ventricular cardiomyopathy and congenital heart disease. Not all patients with NICM underwent cardiac magnetic resonance imaging and myocardial biopsy. Since the diagnosis of tachycardia-induced cardiomyopathy (TIC) would be difficult before the ablation, TIC was defined as improvement in LVEF of > 50% 6–12 months after catheter ablation among the patients with NICM [15]. Patients underwent blood examination, transthoracic echocardiography, and electrocardiogram-gated contrast-enhanced CT scanning before undergoing ablation. Written informed consent was obtained from all patients before enrollment. The protocol was performed in accordance with the Helsinki Declaration and was approved by the Human Investigations Committee of the Hamamatsu University School of Medicine (#20–321).

### Electrophysiological study and catheter ablation

**Fig 1:**
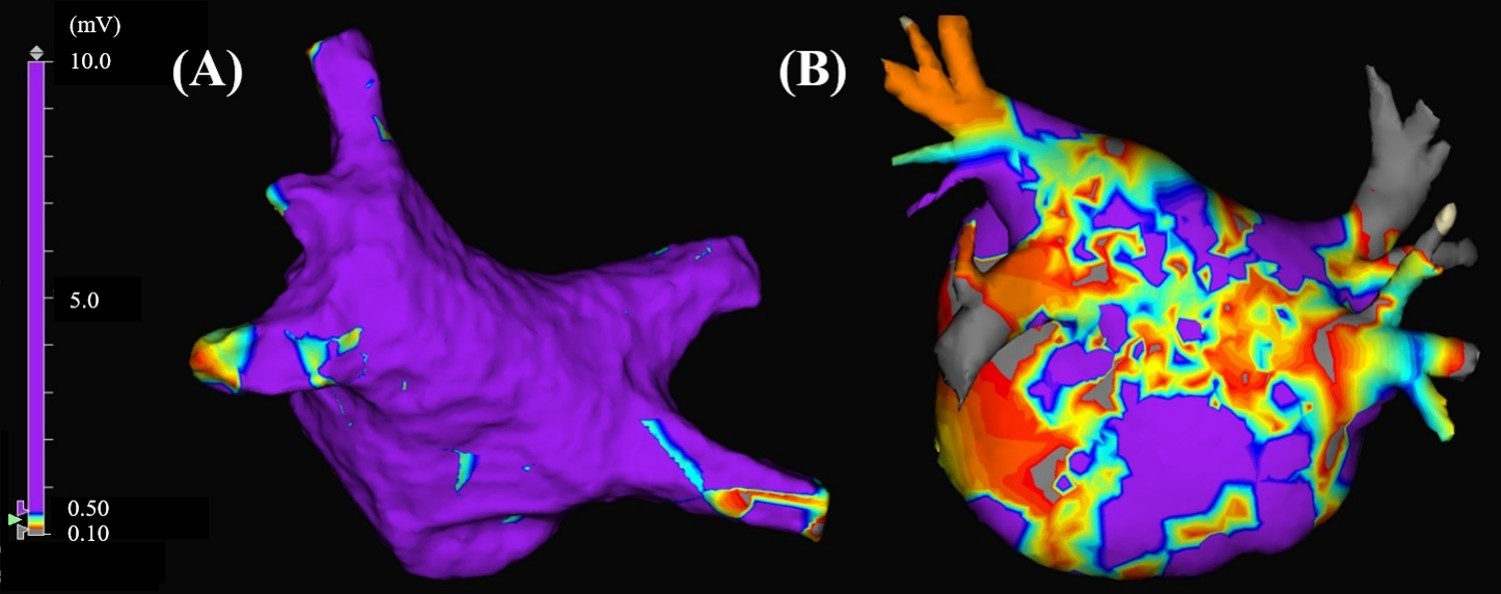
Examples of voltage maps of patients without (A) and with (B) low-voltage zones in the left atrium (posterior–anterior view).

All patients used regular oral anticoagulation at the time of ablation; rivaroxaban, apixaban, and edoxaban treatments were omitted on the morning of the procedure; however, antiarrhythmic drugs prescribed before the ablation were continued. The procedure was performed under intravenous sedation with midazolam and dexmedetomidine. Respiration was supported using adaptive servo-ventilation. Intravenous heparin was administered to maintain an activated clotting time of 300–400 s throughout the procedure. Electro-anatomical mapping was acquired in sinus rhythm or atrial pacing. If AF was observed at the beginning of the ablation procedure, PVI was performed before voltage mapping. When sinus rhythm restoration during PVI was not achieved, electrical cardioversion was performed after PVI and then voltage mapping during sinus rhythm or atrial pacing was performed. The patients with immediate recurrence of AF were excluded from this study because voltage mapping during sinus rhythm or atrial pacing was impossible. Electro-anatomical voltage maps of the LA were created using a 20-pole circular mapping catheter (Optima, Abbott, St Paul, MN, or LASSO, Biosense Webster, Diamond Bar, CA, or Libero, Japan Lifeline, Tokyo) with 3-D electro-anatomical mapping systems (Navx-Ensite Velocity, Abbott, or CARTO 3, Biosense Webster). Mapping points were acquired to fill all color gaps on the entire left atrial surface under the interpolation color threshold of 5 mm in CARTO and 7 mm in Ensite. The detailed settings in CARTO were as follows: auto-annotation algorithms of wave front annotation, tissue proximity indication of off, map consistency of automatic. The detailed settings in Ensite system were as follows: interior projection of 7, exterior projection of 7, and use best duplicate. In both mapping systems, the LVZs were defined as sites with a peak-to-peak electrogram amplitude of < 0.50 mV and extending > 5.0 cm^2^. Areas were measured manually on voltage mapping (Fig 1) [16]. The ablated areas in the initial session were excluded from the voltage analysis in patients who experienced prior catheter ablation of AF.

PVI was performed in all patients who underwent AF procedures *de novo* using open-irrigated radiofrequency catheters, cryoballoons (Arctic Front Advance, Medtronic), or laser balloons (HeartLight, CardioFocus). PVI was confirmed in patients with repeated procedures, and the PV was re-isolated if PV reconnection was observed. Additional ablation, consisting of LA posterior box isolation, superior vena cava isolation, and/or ablation of the spatio-temporal dispersion area, was performed at the operator’s discretion.

### Statistical analysis

Continuous variables were expressed as mean ± standard deviation or median (interquartile range [IQR]). Comparisons between two groups were tested using an unpaired t-test or the Mann–Whitney U test. All categorical variables were presented as numbers and percentages for each group and were compared using the chi-square test or Fisher’s exact test. Univariate analysis of patient characteristics was used to compare patients with and without LVZs. A backward stepwise multivariate logistic regression analysis was performed to detect any independent, significant predictors (reported as odds ratios [ORs] with 95% confidence intervals [CIs]). Variables, including multivariable Cox proportional hazard models, were those that achieved statistical significance (*P* < 0.05) or that were close to significance (*P* < 0.1) in the univariable analysis. In these models, LA volume was divided into 2 groups based on median value. AF recurrence free survival curves were constructed using the Kaplan–Meier method and the survival curves were compared using log-rank test. Statistical significance was defined as *P* < 0.05. All statistical analyses were performed using SPSS version 27.0 (IBM, Armonk, NY, USA). Graphs were compiled with Prism 7.03 (GraphPad, La Jolla, CA, USA).

## Results

### Subject characteristics

A total of 359 patients were enrolled in this study. Baseline characteristics are presented in Table 1. The patients included 73 (20.3%) females, and the mean age was 63.7 ± 10.8 years. There were 149 (41.5%) patients with persistent AF. NICM was observed in 26 patients (7.2%); 11 patients (3.1%) with DCM, 13 patients (3.6%) with HCM, one patient (0.3%) with cardiac amyloidosis, and one patient (0.3%) with valvular heart disease. A prior history of PVI was present in 37 patients (10.3%).

**Table 1:** Patients’ characteristics.

	All	LVZs group	Non-LVZs group	P-Value
	n = 359	n = 51	n = 308	
Demographics				
Age, years	63.7 ± 10.8	67.9 ± 9.9	63.0 ± 10.8	0.003
Female, n (%)	73 (20.3)	17 (33.3)	56 (18.2)	0.013
Body mass index, kg/m^2^	24.6 ± 4.1	24.1 ± 4.0	24.6 ± 4.1	0.411
Current smoker, n (%)	39 (10.9)	4 (7.8)	35 (11.4)	0.454
Use of alcohol, n (%)	174 (48.5)	21 (41.2)	153 (49.7)	0.261
Persistent AF, n (%)	149 (41.5)	35 (68.6)	114 (37.0)	< 0.001
History of prior PVI, n (%)	37 (10.3)	13 (25.5)	24 (7.8)	< 0.001
Clinical characteristics				
HT, n (%)	188 (52.4)	37 (72.5)	151 (49.0)	0.002
DM, n (%)	124 (34.5)	22 (43.1)	102 (33.1)	0.163
Dyslipidemia, n (%)	96 (26.7)	12 (23.5)	84 (27.3)	0.576
Heart failure, n (%)	87 (24.8)	26 (51.0)	61 (20.3)	< 0.001
DCM, n (%)	11 (3.1)	6 (11.8)	5 (1.6)	0.002
HCM, n (%)	13 (3.6)	5 (9.8)	8 (2.6)	0.025
OMI, n (%)	14 (3.9)	2 (3.9)	12 (3.9)	1.000
Medications				
ACE-Is / ARBs, n (%)	125 (35.6)	21 (41.2)	104 (34.7)	0.369
Beta-blocker, n (%)	215 (59.9)	37 (72.5)	178 (57.8)	0.046
AADs, n (%)	271 (75.5)	39 (76.5)	232 (75.3)	0.860
Class I, n (%)	149 (41.5)	16 (31.4)	133 (43.2)	0.113
Class III, n (%)	32 (8.9)	8 (15.7)	24 (7.8)	0.105
Class IV, n (%)	106 (29.5)	20 (39.2)	86 (27.9)	0.101
Laboratory data				
Hb, g/dL	14.2 ± 1.6	13.6 ± 1.7	14.3 ± 1.6	0.005
eGFR, mL/min/1.73 m^2^	64.4 ± 16.9	57.0 ± 16.5	65.6 ± 16.7	0.001
NT-proBNP, pg/mL	232.0 (66.0–569.5)	604.0 (419.0–1042.0)	162.0 (55.0–474.0)	< 0.001
Echocardiography				
LVEF, %	62.7 ± 10.6	60.7 ± 12.4	63.0 ± 10.2	0.142
LAD, mm	38.8 ± 7.0	42.1 ± 6.8	38.2 ± 7.0	< 0.001
LVDd, mm	47.7 ± 5.9	48.0 ± 6.3	47.7 ± 5.8	0.678
LVDs, mm	31.4 ± 6.4	32.0 ± 7.0	31.2 ± 6.3	0.431
Computed Tomography				
LA volume, mL	122.6 ± 66.4	153.6 ± 46.4	117.7 ± 67.8	< 0.001

Data are presented as mean ± SD, median (IQR), or number (%). Abbreviations: AF, atrial fibrillation; PVI, pulmonary vein isolation; HT, hypertension; DM, diabetes mellitus; DCM, dilated cardiomyopathy; HCM, hypertrophic cardiomyopathy; OMI, old myocardial infarction; ACE-Is, angiotensin-converting enzyme inhibitors; ARBs, angiotensin receptor blockers; AADs, antiarrhythmic drugs; Hb, hemoglobin; eGFR, estimated glomerular filtration rate; NT-proBNP, N-terminal prohormone B-type natriuretic peptide; LVEF, left ventricular ejection fraction; LAD, left atrial diameter; LVDd, left ventricular end-diastolic diameter; LVDs, left ventricular end-systolic diameter; LA, left atrium.

#### Features of patients with LVZs

Within the entire cohort, 51 patients (14.2%) had LVZs. A mean size of LVZs was 16.3 ± 16.9 cm^2^. When we divided the LA into four regions; anterior, septal, posterior, and bottom, the extent of areas was as follows; 5.3 ± 6.4 cm^2^ in anterior wall, 4.0 ± 5.4 cm^2^ in septal wall, 5.2 ± 7.4 cm^2^ in posterior wall, and 1.8 ± 2.9 cm^2^ in bottom.

Patients with LVZs were older (*P* = 0.003) and predominantly female (*P* = 0.013) with a higher prevalence of persistent AF (*P* < 0.001) and the use of beta-blockers (*P* = 0.046) than those without LVZs. Concomitant hypertension (*P* = 0.002), heart failure (*P* < 0.001), DCM (*P* = 0.002), and HCM (*P* = 0.025) were higher in patients with LVZs than in those without. In contrast, the prevalence of OMI (old myocardial infarction) did not differ between the two groups. Although there was no significant difference in medication use between patients with and those without LVZs, laboratory testing showed that hemoglobin levels (*P* = 0.005) and estimated glomerular filtration rate (*P* = 0.001) were lower, while N-terminal-prohormone B-type natriuretic peptide levels (*P* < 0.001) were higher in patients with LVZs than in those without LVZs. The LVEF did not significantly differ between the two groups. Conversely, patients with LVZs had larger LA volumes, measured using CT (*P* < 0.001), and longer LA diameters, measured using echocardiography (*P* < 0.001), than those without LVZs.

Multivariate logistic regression analysis revealed that age (OR 1.060; 95% CI 1.022–1.101, *P* = 0.002), female sex (OR 2.978; 95% CI 1.340–6.615, *P* = 0.007), DCM (OR 8.341; 95% CI 1.381–50.372, *P* = 0.021), HCM (OR 5.044; 95% CI 1.314–19.363, *P* = 0.018), persistent AF (OR 4.188; 95% CI 1.928–9.100, *P* < 0.001), and larger LA volume (OR 3.215; 95% CI 1.378–7.502, *P* = 0.007) were independently associated with the presence of LVZ (Table 2).

**Table 2:** Predictors of LVZs.

	Univariate		Multivariate	
	Odds Ratio (95% CI)	P-value	Odds Ratio (95% CI)	P-value
Age	1.051 (1.017–1.087)	0.003	1.060 (1.022–1.101)	0.002
Female	2.250 (1.174–4.311)	0.015	2.978 (1.340–6.615)	0.007
DCM	8.080 (2.368–27.573)	0.001	8.341 (1.381–50.372)	0.021
HCM	4.076 (1.278–12.998)	0.018	5.044 (1.314–19.363)	0.018
Persistent AF	3.723 (1.973–7.025)	< 0.001	4.188 (1.928–9.100)	< 0.001
LA volume of >113 ml	4.554 (2.189–9.478)	< 0.001	3.215 (1.378–7.502)	0.007
LV ejection fraction	0.981 (0.956–1.007)	0.143		

Abbreviations: CI, confidence interval; DCM, dilated cardiomyopathy; HCM, hypertrophic cardiomyopathy; AF, atrial fibrillation; LA, left atrium.

In addition, when we defined tachycardia-induced cardiomyopathy (TIC) as improvement in LVEF after catheter ablation, TIC was observed in four patients (1.1%) which was originally classified as DCM (“true DCM”: 7 [1.9%] patients) during the mean follow up period of 10.6 months. In this setting, true DCM was higher in patients with LVZs (4 [7.8%] vs. 3 [1.0%], *P* = 0.009), however, TIC was not (2 [3.9%] vs. 2 [0.6%], *P* = 0.098). Also, multivariate logistic regression analysis demonstrated that true DCM was still an independent predictor of LVZs (OR 31.661; 95% CI 3.108–332.519, *P* = 0.004).

We performed a sub-analysis regarding the type of AF. Out of 210 patients with paroxysmal AF, 16 patients had LVZs. Age (OR 1.079; 95% CI 1.001–1.1163, *P* = 0.046), female sex (OR 4.427; 95% CI 1.317–14.880, *P* = 0.016), DCM (OR 29.532; 95% CI 1.221–714.519, *P* = 0.037), and HCM (OR 13.304; 95% CI 2.513–70.428, *P* = 0.002) were independent predictors for the presence of LVZs in patients with paroxysmal AF. On the other hand, voltage mapping showed LVZs in 35 of 149 patients with persistent AF. Age (OR 1.054; 95% CI 1.009–1.101, *P* = 0.018) and LA volume of > 138.9 ml (OR 2.944; 95% CI 1.220–7.103, *P* = 0.016) independently predicted the presence of LVZs in patients with persistent AF.

**Fig 2:**
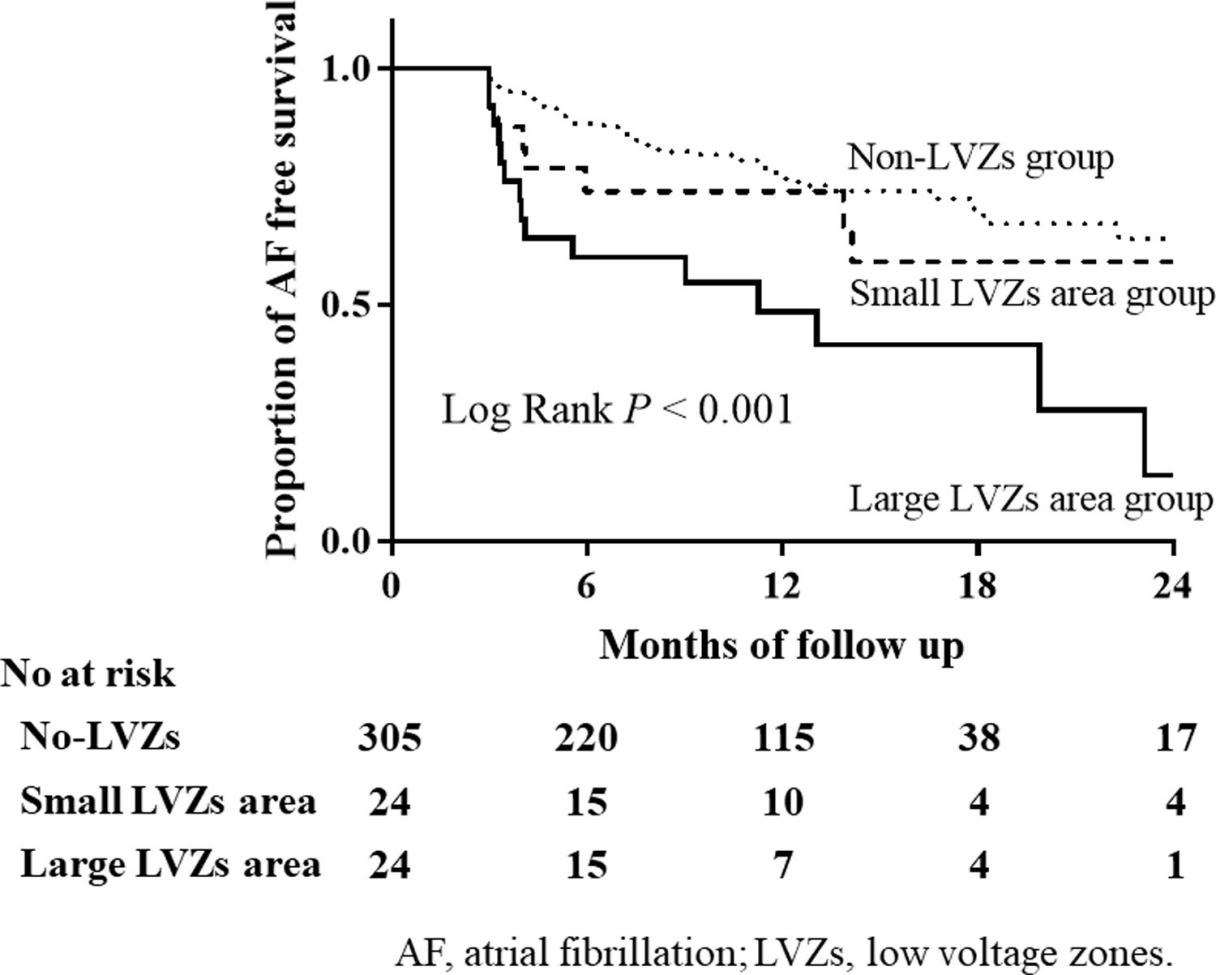
Kaplan-Meier curve of freedom from AF recurrence.

When we divided the study subjects into 3 groups: patients without LVZs, those with small LVZs, and those with large LVZs according to their median area of LVZs, Kaplan-Meier curve showed that patients with large LVZs had an increased risk of AF recurrence after the ablation compared with patients with small LVZs and those without LVZs (*P* < 0.001 by log rank test, Fig 2).

## Discussion

The main findings of this study were that older age, female sex, DCM, HCM, and larger LA volumes were independent pre-procedural predictors of LVZs.

### Clinical impact of LVZ and importance of pre-procedural predictors for LVZ

PVI is the cornerstone of ablation treatment for AF because most triggers of AF originate from PVs [17]. However, some patients experience AF recurrence despite durable PVI. One possible explanation is atrial remodeling, which results in non-PV foci. LVZs could indicate myocardial fibrosis and scarring, which may play important roles in the arrhythmogenesis of AF. Studies have shown that the presence of LVZ is associated with worse outcomes after AF ablation [18]. Targeting LVZ beyond PVI is a logical method to suppress the recurrence of AF in patients with LVZ. In fact, the beneficial effect of LVZ ablations beyond PVI on the maintenance of sinus rhythm after ablation has been demonstrated [13, 14, 19]. However, identifying LVZs requires electrophysiological analysis with intracardiac electrodes. The complete additional ablation for LVZs using balloon-based devices is challenging. Therefore, predicting the presence of LVZs before the procedure enables the planning of suitable procedural approaches such as using a radiofrequency ablation catheter.

### Pre-procedural predictors for LVZs

Although late gadolinium-enhanced cardiac magnetic resonance imaging can detect LA fibrosis, and the extent of the late gadolinium enhancement area correlates with a high recurrence rate of AF after ablation [20], most hospitals would not be able to perform routine cardiac magnetic resonance imaging before the ablation. Age, sex, and LA volume are the components of assessment tools, such as APPLE, modified APPLE, and DR-FLASH scores, which reportedly predict LVZs [18, 21, 22]. Our findings also revealed that age, female sex, and a larger LA volume were independent pre-procedural predictors for LVZs in line with previous reports. To the best of our knowledge, this is the first study to demonstrate that DCM and HCM are independent predictors of LVZs.

AF is known as an intercurrent arrhythmia with other cardiovascular diseases. The incidence of AF is 6–13% after myocardial infarction [23–25], 22.5% in HCM [26], and 20% in DCM with heart failure (HF) [27]. In our study, patients with DCM or HCM but not with OMI had a higher prevalence of LVZs compared to those without. It has been reported that even HF patients without AF present LVZs and a silent area in the atrium [28]. In addition, patients with HCM presenting with AF have a high burden of left atrial fibrosis on magnetic resonance imaging, indicating atrial remodeling [29]. The differences among DCM, HCM, HF, TIC, and OMI could be dependent on the damaged segment; the damage of the whole heart segment is due to DCM, HCM, and HF; on the other hand, the damage for OMI predominantly localizes to the territory of coronary arteries that was mainly in ventricles not in atriums. In terms of TIC, LA may not be permanently affected since TIC is a reversible condition of left ventricular systolic function.

Our findings showed that DCM and HCM were independent predictors for the presence of LVZs not in patients with persistent AF but in those with paroxysmal AF. One possible speculation was the progression of LA remodeling. Since LA is normally not so diseased in patients with paroxysmal AF, concomitant DCM or HCM plays an important role to form LVZs.

### Limitations

This study has several limitations. First, this was a single-center, retrospective study. The small sample size limited the power of the study. Second, there is no randomized controlled trial to prove the beneficial impact of an additional ablation targeting LVZs on the maintenance of sinus rhythm after ablation, although this has been shown in several non-randomized studies. Third, it has been reported that the detected voltage mapping during pacing rhythm could differ according to the pacing sites or cycle length [30]. In our study, we did not consider these factors. Forth, 20-pole circulatory mapping catheter was used in this study. It was reported that the detection sensitivity of LVZs could be affected by the type of catheter used for voltage mapping [31] and the extent of LVZs detected by 20-pole circular mapping catheter could be measured smaller by multi-electrode high-density mapping catheter [32] that could overestimate the presence and extent of LVZs. Therefore, prospective multicenter studies are needed to resolve these limitations and further validate our results.

## Conclusion

Patient age, female sex, DCM, HCM, persistent AF, and LA volume may predict the presence of LVZs and could be useful in selecting an appropriate ablation strategy for AF before the ablation procedure.

## Supporting information

S1 Data (CSV)
